# Equity, Diversity, Inclusion and Accessibility in Pharmacy Education: A Scoping Review

**DOI:** 10.3390/pharmacy14030076

**Published:** 2026-05-20

**Authors:** Mirey Alfarah, Ivy Kan, Marie A. Vander Kloet

**Affiliations:** 1 Centre for Pharmacy, Department of Global Public Health and Primary Care, University of Bergen, 5009 Bergen, Norway; 2 Center for Medical Education, The Medical Faculty, University of Bergen, 5009 Bergen, Norway; 3 Department of Infectious Diseases and Public Health & Primary Care, Leiden University Medical Center, 2333 ZA Leiden, The Netherlands; i.kan@lumc.nl; 4 Department of Education, University of Bergen, 5015 Bergen, Norway; marie.vander.kloet@uib.no

**Keywords:** pharmacy education, inclusion, equity, diversity, accessibility, teaching strategies, curriculum, faculty development, inclusive pharmacy care

## Abstract

Preparing pharmacists to serve diverse populations requires a meaningful integration of equity, diversity, inclusion, and accessibility (EDIA) within pharmacy education, yet such integration remains uneven and insufficiently understood. This scoping review aimed to examine how EDIA is addressed across faculty development, curriculum content, and teaching strategies in pharmacy education. Following the guidance of the Joanna Briggs Institute, we searched six databases (Embase, Medline, APA PsycINFO, CINAHL, ERIC, and Web of Science) for studies published between 2014 and 2025. After screening 3031 records, 86 studies met the inclusion criteria. Most studies focused on curriculum (48/86) and teaching strategies (35/86), while very few examined faculty development (3/86). Research was heavily concentrated in the United States of America and relied predominantly on survey-based methods. EDIA topics were often addressed in isolation with a strong emphasis on intercultural communication and limited attention to areas such as disability, migration, and socioeconomic status. Intersectional approaches were rare. Overall, EDIA in pharmacy education appears fragmented and commonly implemented as standalone initiatives rather than integrated across programs. These findings highlight important gaps in faculty development in pharmacy education, methodological diversity, and global representation, and they point to persistent structural gaps and the need to strengthen faculty development initiatives specific to pharmacy education and to move beyond isolated initiatives toward a sustained, program-level integration of EDIA.

## 1. Introduction

Equity, diversity, inclusion and accessibility (EDIA) are frequently named as core commitments and values for public higher education institutions. Engaging in EDIA practices in higher education can demonstrate to students that institutions are committed to fostering participation and strengthening a sense of belonging for all learners, which can, in turn, help create a safer educational environment. Incorporating EDIA efforts in higher education has demonstrated various benefits, including improvements on learning outcomes such as active-thinking skills, intellectual engagement, and motivation. Additionally, these efforts have been associated with students’ self-reported empathy, civic awareness, and perceptions of democratic engagement [1].

EDIA concepts are defined in multiple ways across policy, research, and educational contexts. To ensure consistency and to support a global perspective, this paper adopts definitions primarily based on internationally recognized frameworks developed by the United Nations (see Table 1).

For pharmacy education, embedding EDIA principles and practices is essential. Pharmacists contribute to the safe and effective use of medicines. Historically, pharmacists had a more technical focus, working in all levels of pharmaceutical industry, from drug discovery and development to drug manufacturing, drug registration and pharmacovigilance. They provided similar services in hospitals and community pharmacies—quality control of prescriptions, on-site production (e.g., re-formulating drugs for use in children), monitoring the use of antibiotics to prevent antibiotic resistance—and they filled essential functions in drug regulatory agencies. Over the last 25 years, however, with the emergence of patient-centered medicine, there has been a widening in the scope of practice for pharmacists. Pharmacists are now taking on new roles [5]: counseling patients in the safe use of medicines, on how to treat minor ailments, and on increasing adherence to prescribed treatments and vaccinations, and collaborating with physicians and other health personnel to optimize treatment in municipal and hospital care. A critical lack of general practitioners and other health care personnel has also led to calls for better use of the advanced drug knowledge of pharmacists and for pharmacists to take on new responsibilities.

Pharmacy programs are responsible for preparing future pharmacists to work effectively with diverse patient populations and to contribute to health equity [6,7]. This requires not only strong clinical knowledge but also the ability to communicate across differences and to recognize barriers that may limit access to pharmacy care for marginalized groups [8]. EDIA-informed pharmacy education can therefore support graduates in recognizing and addressing barriers to care, adapting communication and services to patient needs, and contributing to equitable health outcomes. It is also relevant to interprofessional practice, as pharmacists increasingly work in collaborative teams where inclusive communication, awareness of professional hierarchies, and advocacy for underserved patients are central to safe and person-centered care.

At the same time, the increasing diversity of student pharmacists [9] highlights the importance of faculty development and the need to support educators in creating inclusive learning environments in pharmacy education. More broadly, pharmacy education is shaped by structural inequalities that affect higher education as a whole, including barriers related to study formats and the disproportionate attrition of marginalized students [10]. Embedding EDIA practices in pharmacy education is therefore necessary to support inclusive learning environments and to better prepare graduates for equitable and inclusive pharmacy practice.

Within pharmacy education, several reviews have been published that explore key themes related to EDIA. The concept of the hidden curriculum in pharmacy education is examined by Park et al. [11], defining it as the teaching and learning that occur outside the formal curriculum. This hidden curriculum encompasses the knowledge, skills, attitudes, behaviors, values, and beliefs that students are expected to (sub)consciously acquire and accept to be successful in pharmacy programs. While it plays a significant role in shaping professional identity, it can also negatively impact interprofessional practice, professional norms, ethics, and ultimately, patient outcomes. This occurs through the transmission of implicit norms, values, and behaviors that may not always align with evidence-based care or professional guidelines. A scoping review conducted by Ho et al. [12] that focuses on evidence-based tools and strategies for developing cultural competence highlights that the most common structures for students were required courses, elective courses, and study abroad experiences, and the most commonly employed activities were lectures, reflections and discussions. They concluded that integrating these activities systematically throughout the curriculum could enhance their effectiveness. Lastly, a review and categorization of published educational research on diversity in pharmacy schools has been conducted by Bush et al. [13]. Their review showed that contemporary drivers of change are shaping diversity-focused research in pharmacy education, and the authors emphasize that greater attention is needed to the research foci, targets, and resulting recommendations. They note that although numerous calls for diversity-related research have been made, the scholarship in this area remains limited, leading them to conclude that diversity has not yet been adequately addressed and that further commitment is required. While these reviews each address important EDIA-related themes, they focus on specific aspects of this work (e.g., hidden curriculum, cultural competence). What is missing is a comprehensive overview of how EDIA is embedded within pharmacy education that provides a fulsome picture of research in this area. This gap underscores the need for the present scoping review.

Despite growing recognition of its significance, a comprehensive overview of research that examines EDIA broadly within pharmacy education is absent. Including the aforementioned reviews, research in this field often focuses on very specific aspects of EDIA in pharmacy education, highlighting the need for a comprehensive review. This paper seeks to address this gap and provides a more complete review of the literature, offering valuable insights for pharmacy educators, educational developers or higher education researchers. This review can support pharmacy educators in gaining insights into EDIA-related research to inform their teaching strategies. Educational developers can use it as a resource to implement inclusive teaching practices, while pharmacy education researchers can utilize this mapping to plan and design studies exploring specific aspects of EDIA in pharmacy education. The objective of this scoping review is to map and synthesize existing literature which investigates EDIA in pharmacy education, focusing on three broad areas: faculty development, curriculum content, and teaching strategies. Specifically, this review summarizes how this research is conducted and what EDIA areas are addressed in the research.

### Review Question

Recognizing the growing importance of integrating EDIA principles in pharmacy education and health care [14], this review aims to systematically map and summarize existing research and potential knowledge gaps in this area. The key research question which guides this review is *What literature exists pertaining to EDIA in pharmacy education that is specifically focused on faculty development, curriculum content, and teaching strategies?*

To address this overarching question, this paper considers the following subquestions:

- What faculty development programs related to EDIA are used in pharmacy programs? How are these programs designed and implemented, and what specific EDIA focus do they address?
- What curriculum content and development processes related to EDIA are used in pharmacy programs? What specific aspects of EDIA are emphasized?
- What teaching strategies related to EDIA are used in pharmacy programs? Do these strategies target particular aspects of EDIA?

This scoping review was developed following the guidelines of the Joanna Briggs Institute (JBI) and the JBI Scoping Review Methodology Group [15]. A protocol for this scoping review was developed a priori and published in BMJ Open [16], detailing the methodology and planned analyses.

By applying the JBI scoping review methodology, this paper contributes to the understanding of how EDIA principles are integrated into pharmacy education and provides a foundation for future research in this area.

## 2. Materials and Methods

This review was performed in accordance with the PRISMA Extension for Scoping Reviews (PRISMA-ScR) guidelines, and the completed PRISMA-ScR checklist is provided in the Supplementary Materials [17].

### 2.1. Search Strategy

To cover both medical and educational literature, the literature searches were run in the medical and health sciences databases Embase (Ovid), Medline (Ovid), APA PsycINFO (Ovid) and CINAHL (Ebsco), in the educational database ERIC (Ebsco), and in the multidisciplinary database Web of Science (see Supplementary Materials). Both subject headings and free text terms were used to cover the concepts of pharmacy education and EDIA. The publication date was limited to 2014 onwards due to our interest in recent interventions and activities which have taken place during a period in which many, if not all, Global North academic institutions have developed and implemented policies or statements related to EDIA. Earlier equity work may have focused on single equity issues (e.g., gender balance in study programs). Additionally, through preliminary searching, we found that much of the literature is from the last 5 years, suggesting that the majority of this scholarship is relatively new (hence, a 10-year history is sufficient for providing an accurate summary of this work). No national/location restrictions were applied. Test searches were run in November 2024, and the search strategy was peer-reviewed by an academic librarian.

A full search was completed in March 2025. Retrieved records were then imported to the reference software EndNote 2025. Deduplication using ASySD 0.4.6 was conducted prior to exporting data from EndNote 2025 [18]. Additional identified duplicates were reviewed manually in the review software (Covidence) prior to beginning the review.

### 2.2. Inclusion and Exclusion Criteria

The scoping review used the PCC (Population, Concept, Context) framework to establish inclusion and exclusion criteria. Studies from 2014 to 2025 were included if they met the following criteria.

#### 2.2.1. Population

Papers focusing on individuals involved in pharmacy education were included, specifically the following:

- Faculty members and educators in pharmacy programs.
- Students at undergraduate, graduate, or postgraduate levels in pharmacy programs.
- Pharmacy education leaders, administrators and stakeholders involved in curriculum development or teaching strategies related to EDIA.

Papers focusing on populations entirely outside of pharmacy education, such as those from medical or nursing education, were excluded. Some studies which included populations from multiple disciplines were included (e.g., pharmacy and nursing students in a course that is jointly operated between two study programs).

#### 2.2.2. Concept

Studies were included if they examine the integration of EDIA principles in pharmacy education through faculty development, curriculum content, or teaching strategies. Faculty development initiatives must explicitly address EDIA in their design or implementation with a focus on topics such as gender, race, ethnicity, disability, intercultural competence, or other social identities, and they must be specific to pharmacy education (or pharmacy and other disciplines). Studies on curriculum content were considered if they explore the integration of EDIA-related themes, including health inequities, intercultural communication, decolonization, or inclusive learning approaches. Teaching strategies must involve EDIA-focused pedagogical methods, such as experiential learning, simulation-based instruction, or community-engaged approaches within pharmacy education.

Studies were excluded that did not directly engage with EDIA principles in a meaningful way, only mention them peripherally, or focus on unrelated methodologies or topics. There are three larger and specific areas that we excluded because they were either outside the scope of the research questions or did not engage directly with EDIA. These are addressed further in the discussion and recommendations for future research. First, we excluded studies undertaken to prepare for curriculum change (even if they engaged with EDIA directly). These studies, while related to curriculum work, are not directly connected to the scoping review question. Importantly, although the research may be equity focused, it did not include any analysis of EDIA work that has been developed and implemented in any curriculum. Second, we excluded research focused on Mental Health First Aid that did not engage critically with ableism. Mental Health First Aid is a very common inclusion in formal and co-curriculum for all health professionals (including pharmacists), but this training does not typically focus on EDIA in its implementation. Third, we excluded formal or co-curricular training activities which used the language of intercultural competence or learning but did not take up EDIA principles—for example, survey research related to students’ participation in ‘preparation for semester abroad’ workshops which covered many topics (expenses, vaccinations, responsibilities, cultural differences or norms) but did not entail specific learning activities, assessments or outcomes related to EDIA. These three key areas were excluded to ensure the research questions are addressed and the scoping review is directly focused on EDIA in pharmacy education.

#### 2.2.3. Context

Eligible studies must take place within formal pharmacy education settings across academic, professional, or institutional contexts. This includes pharmacy programs offered at undergraduate, graduate, or postgraduate levels as well as faculty development workshops and structured courses aimed at pharmacy educators. Studies were included if they assess EDIA integration in various teaching and learning formats, such as standalone or embedded courses, online or in-person delivery, and structured faculty training initiatives. Co-curricular activities that were not requirements were excluded.

Studies must be published in English or at minimum have an abstract and keywords available in English. Research focusing on education outside formal pharmacy programs, such as public health campaigns or general workforce training, was excluded. Studies focusing solely on preliminary curriculum exploration (e.g., needs assessments or curriculum mapping without implementation or evaluation) were also excluded. Additionally, studies that do not explicitly assess the implementation or evaluation of EDIA-related content in pharmacy education were excluded. Only empirical studies were included; commentaries, editorials, recommendation or discussion papers and other gray literature were excluded. The exclusion of gray literature reflects this scoping review’s explicit focus on empirical research.

### 2.3. Study Selection and Data Extraction

**Figure 1:**
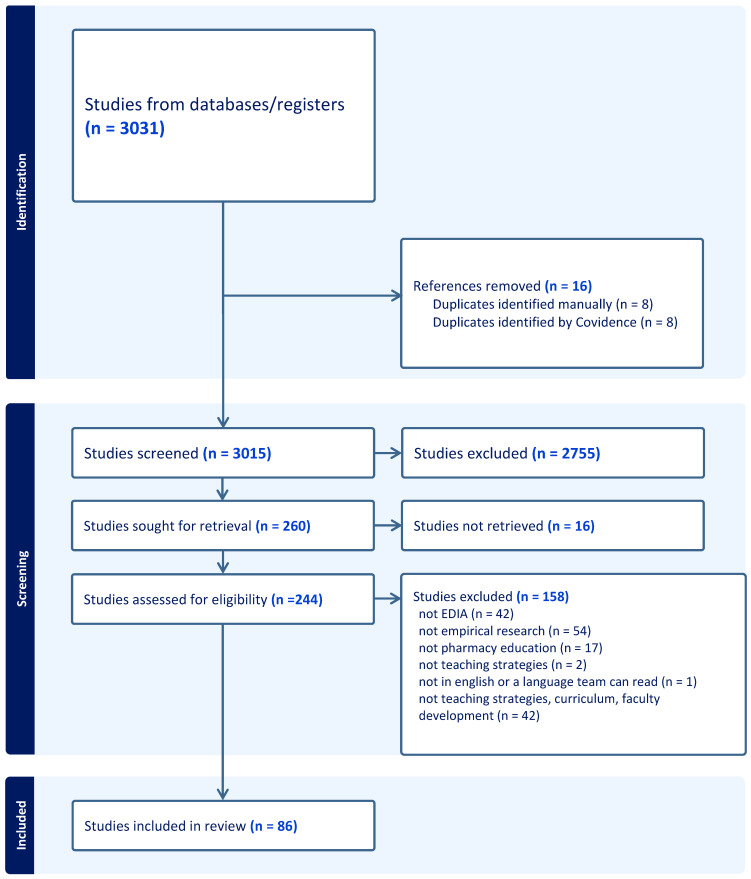
PRISMA flow diagram.

Following the completion of the search and removal of all duplicates, study screening was completed. All screening was managed using Covidence software. All records’ titles and abstracts were screened independently by two researchers with discrepancies resolved a third researcher or, if needed, consultation among all researchers. Full-text screening used the same process, ensuring that only eligible studies were included. At the full-text screening stage, records were excluded if they did not include EDIA (*n* = 42); were not empirical research (e.g., opinion pieces, letters to journal editors) (*n* = 54); were not about pharmacy education (*n* = 17); were not about teaching strategies, curriculum or faculty development (e.g., recruitment programs) (*n* = 44); or were not in English or another language the team could review (*n* = 1). All included or excluded studies can be reviewed in the PRISMA flow diagram in Figure 1.

Data extraction was conducted systematically using a predefined extraction tool (see Supplementary Materials) to ensure consistency and accuracy. The tool was developed by two researchers (MA and IK) and piloted by a larger research team working in pairs on eight studies selected by IK. The piloting phase enabled the refinement of the tool and addressed ambiguities prior to undertaking full extraction. At this stage, the extraction tool was revised, and refinements were made to the research questions.

For data extraction, two researchers extracted information from each included study, capturing bibliographic details, study characteristics, and findings related to EDIA in faculty development, curriculum content, and teaching strategies. Extracted data were reviewed for consistency, and all discrepancies between reviewers were resolved before beginning data analysis. Extracted data were analyzed descriptively using frequencies and thematic categorization aligned with the review questions. Consistent with scoping review methodology, no formal critical appraisal of included studies was conducted.

## 3. Results

A total of 3031 were identified in the initial search. Following screening, 260 studies were included for full-text review. A further 16 studies were removed due to incomplete or partial records (e.g., conference abstracts only). From the remaining 244 records, 86 met the inclusion criteria and are included in this scoping review (see Figure 1).

A total of 86 peer-reviewed studies published between 2014 and 2025 were included in the review. Nearly all were available in English (85/86). The literature was heavily concentrated geographically with the majority conducted in the United States (68 studies), followed by Australia (6), Canada (5), the United Kingdom (3), and New Zealand (2), with isolated contributions from Germany, Lebanon, Malaysia, Saudi Arabia, and Brazil. When grouped by region, most studies originated from North America with smaller clusters in Oceania, Europe, and Asia.

In terms of focus areas, most studies addressed curriculum content (48/86), followed by teaching strategies (35/86), while studies of faculty development were infrequent (3/86). Study designs were dominated by surveys (59) with smaller numbers of reviews (11), mixed-methods studies (7), qualitative designs (3), observational studies (2), and single examples of quasi-experimental, case study, and other approaches. The primary populations studied were students: graduate or postgraduate students were included in 70 studies and undergraduate students in 56; only four studies explicitly involved faculty or staff populations.

Participant numbers were extractable from 75 studies. The median sample size was 109 participants (IQR 54–168), ranging from 6 to 1009. Median sample sizes differed somewhat by domain: curriculum-focused studies had a median of 95.5 participants, 134 teaching-strategy studies, and 68 faculty-development studies.

Across the corpus, explicit engagement by researchers with structural concepts such as power, privilege, social location, or bias in relation to their own identities was rare; only five studies acknowledged these elements in their framing or interpretation of the research. Most articles were written solely by pharmacy faculty (56/86), a smaller portion jointly by pharmacy faculty and others health disciplines (23/86), and fewer still by pharmacy faculty with scholars from outside of health fields or community partners (8/86). Among curriculum studies, 26 provided recommendations, while 12 did not. Among teaching-strategy studies, 17 provided recommendations, and 9 did not. Faculty-development studies were too few and sparsely coded to support any meaningful quantitative interpretation of recommendations or authorship.

### 3.1. EDIA Focus Areas

**Figure 2:**
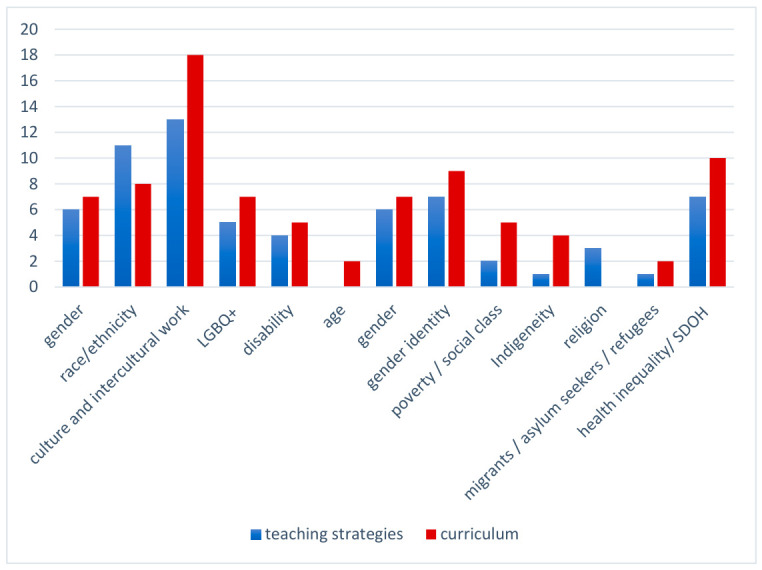
EDIA focus areas.

This scoping review was specifically interested in uncovering what EDIA areas are attended to in the literature. Faculty development studies were too few (*n* = 3) to include in this assessment. Teaching strategies and curriculum studies were assessed in order to review what areas of EDIA are addressed in the research. Most studies (*n* = 52) focused on a single EDIA area (e.g., disability; poverty), while the remaining 34 studies focused on two or more aspects of EDIA (See Figure 2). Common patterns were to focus on two areas together (e.g., religion and intercultural communication; poverty and SDOH)).

Unquestionably, pharmacy education research on EDIA focuses on culture and intercultural work (umbrella category including intercultural communication, intercultural competence, etc.). Notably, research on intercultural activities is also a significant research area that was outside of the scope for this review (for limited engagement with EDIA). We noted that some EDIA areas that are often taken together in inclusion work (e.g., gender and sexual diversity) were at times taken up separately in this research. For example, we observed that gender identity and transgender people’s health care needs were often taken up separately and specifically in the research (particularly with attention to medication dosing and communication) (thus, in Figure 2, we identify LGBQ+ and gender identity as separate categories). Additionally, there is work that is highly specific to national contexts (e.g., work in Canada, New Zealand, or Australia to strengthen work on indigeneity in health care as a response to legislation or political action to address colonialization). Significantly, there is very sparse research in several areas including disability, age, religion, poverty and migration status.

Although this research sometimes addressed more than one focus area, it was rare that these studies were informed by an intersectional approach to difference and power. This attention to one aspect of difference (e.g., gender, race, religion) is likely related to the methods used in the research (examined further in Section 3.2). Design of intersectional EDIA work and scholarly analysis using an intersectional lens is more complex, challenging and time demanding. We anticipate pharmacy educators may be engaged in intersectional EDIA activities in their teaching, although research on these efforts may not be undertaken.

Finally, given that most research comes from the USA, much of how EDIA focus areas are addressed reflects the political and social context from the USA. Pharmacy educators from other regions may be more concerned with other EDIA areas (e.g., Europe and migration), but research from outside the USA is exceedingly sparse. In many ways, the EDIA categories that we identified and employed reflect the research included in this scoping review and thus in turn reflect a particular country/region’s focal areas when it comes to EDIA work. The relevance of these categories as a meaningful way to represent EDIA in pharmacy education is thus time and place specific.

### 3.2. Research Objectives and Methods

**Figure 3:**
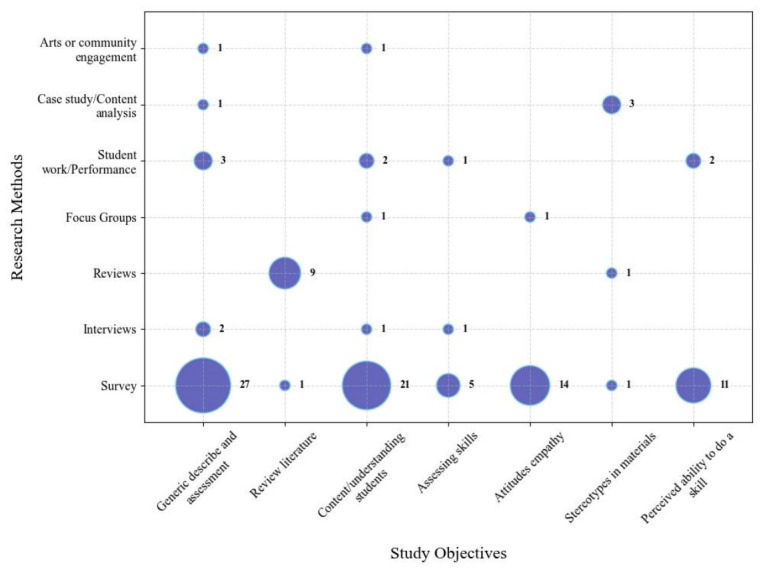
Research objectives and methods.

This scoping review investigated both the objectives and methods used in EDIA-focused pharmacy education research. We were curious both what this research asks (through the objectives) and how it tries to answer and take up these lines of inquiry (through the methods). The relationship between the research objectives and methods is presented in Figure 3.

Most studies (61/86) had a single research objective or two objectives (23/86). Only two studies have more than two objectives. We coded all research objectives, identifying the seven most common types of objectives. The most common objective was to describe and provide a general assessment of a particular practice (teaching strategy or curriculum) (*n* = 30). This was followed by the objective of assessing students’ understanding of a particular content area (*n* = 21). Other common objectives include to evaluated students’ attitudes or empathy (to a marginalized group) after a teaching strategy/curriculum activity (*n* = 15) and to evaluate students’ self-perception of their ability to acquire a specific skill (post-teaching strategy/curriculum activity) (*n* = 15). There were several reviews in our scoping review, the objective of these studies was reviewing a particular content area in pharmacy education (*n* = 10). Additionally, several studies included the objective of assessing the development of a particular skill set in students (*n* = 6) and investigating stereotypes in teaching materials or curriculum (*n* = 5).

To investigate these objectives, we observe key patterns regarding research methods used in these studies. Overwhelmingly, these studies rely on surveys as the sole or primary means of collecting data (69/86). Of these 67 studies, only 13 studies used an additional research method. Other common methods include interviews (*n* = 4), reviewing (scoping, integrative, exploratory) (*n* = 9), focus groups (*n* = 2), analyzing students’ course assignments or performance on assessments (*n* = 8), case studies or content analysis (*n* = 4) and arts-based methods or community engagement methods (*n* = 2). Except for studies that had the primary objective of reviewing literature, the most common research method was the use of surveys.

The objectives and methods of the studies in this scoping review highlight a clear pattern of using surveys to assess primarily if, or to what extent, a teaching strategy or curriculum change ‘works’. The focus on surveys to provide general assessments of teaching strategies and curriculum or to assess students’ understanding suggests narrow scopes of inquiry. These objectives and approaches make both broad and intersectional approaches to EDIA work unlikely. Moreover, the absence of multi-institutional or multi-country studies, longitudinal analyses or detailed mixed-methods approaches limits the scope of research objectives.

## 4. Discussion

### 4.1. Lack of Faculty Development Training in Pharmacy Education

This review shows that there are very few studies focused on faculty development, pharmacy education and EDIA (*n* = 3). Contrastingly, both curriculum content and teaching strategies were widely represented. The limited research on faculty development is a significant gap in EDIA in pharmacy education; we know little about if faculty development activities take place, what faculty development takes place, what impact these activities have on educators and what areas of EDIA are included.

Faculty development research is crucial to inclusive pharmacy education. Faculty play a central role in shaping the learning environment through their teaching practices, feedback, assessment and everyday interactions with pharmacy students. Research in higher education shows that the quality of relationships between students and teachers is closely linked to learning, motivation, and students’ sense of belonging, especially for marginalized groups [19]. Trust plays a key role in these relationships. Students are more willing to participate, ask questions, and take learning risks when they believe their teacher is competent, fair and truly cares about them and their learning [20]. Yet, few faculty development programs focus intensively on EDIA, and even fewer are discipline specific.

Certainly, there may be access to institutional or multidisciplinary EDIA faculty development for pharmacy educators, although this varies considerably nationally and regionally. Structured and discipline-specific EDIA-related faculty development can support educators to develop knowledge and competency on inclusive teaching practices and curriculum development specifically in the context of pharmacy education. Additionally, discipline-specific training can ensure that EDIA is taken up in ways that connect with pharmacy education specific needs. Moreover, EDIA-focused faculty development in pharmacy education could also be best designed to support the many kinds of educators pharmacy students encounter (including academic staff and clinical educators). Meaningful and context-specific training can effectively provide educators with the needed tools to recognize bias, create a safe space for learning, and design learning experiences that are inclusive and responsive to diverse students’ needs [21,22,23]. However, given the paucity of research on this type of faculty development, further inquiry is urgently needed to understand how and in what ways these activities can be designed to support educators and, in turn, impact students’ experiences in pharmacy education.

### 4.2. Geographical Concentration and Policy Context

Similarly concluded by Swidrovich [24], we can clearly see from the results that the evidence base for EDIA in pharmacy education is heavily concentrated geographically in the United States (USA) with comparatively few contributions from other countries/regions. Certainly, there are many possible explanations for the predominance of research from the USA (e.g., size of the higher education system, English language publishing in pharmacy education broadly). What is crucial to consider is the impact of this high concentration of American scholarship on the field of inclusive pharmacy education. Certainly, how EDIA is conceptualized and investigated is place specific; as such, reliance on scholarship that is predominantly from the USA may be challenging for higher education institutions in other countries and regions that must address very different historic and contemporary inequalities, which may frame EDIA principles differently.

Moreover, a global reliance on EDIA scholarship from the USA is particularly concerning. In the USA, the broader political and funding environment for diversity initiatives has shifted, and recent policy actions have signaled constraints on or an outright prohibition of diversity, equity, and inclusion efforts at federal and institutional levels, including changes in compliance expectations and the elimination of or threats to funding tied to EDIA programming [25,26]. Much of the research in this scoping review was motivated by expectations for EDIA work related to institutional, organizational or professional mandates. If EDIA work is deprioritized or outright banned, engagement in this type of work as educators and researchers carries significant risk. For pharmacy educators, many of whom are engaging in EDIA work and research on pharmacy education on top of complex and challenging teaching and research responsibilities, it is likely they will reduce or step back from this type of work.

Given this significant shift in EDIA in higher education in the USA, pharmacy educators, faculty development and higher education scholars both within the USA and internationally will likely quickly observe a significant gap in research about inclusion in pharmacy education. Global reliance on the preponderance of EDIA scholarship from the USA shows how vulnerable this work is. Contributions from scholars globally to EDIA in pharmacy education research are urgently needed both to broaden the field and to ensure that research in this field is not hollowed out. Although certainly many pharmacy educators may face similarly hostile environments for EDIA work to that in the USA, there are many national and regional contexts where pharmacy educators can and do engage in this work; scholarly work is needed from them. Better equipping pharmacy educators who engage in EDIA work to contribute to scholarship on teaching may be possible through or coupled with faculty development on EDIA in pharmacy education.

### 4.3. Fragmented and Isolated: EDIA in Pharmacy Education

The research in this scoping review on EDIA in pharmacy education shows that how EDIA is integrated into pharmacy education is both isolated and fragmented. The empirical research shows that EDIA content was most often delivered as a standalone session, workshop or as an elective course rather than being embedded across the curriculum or adopted widely in teaching methods. This pattern aligns with observations in the broader higher education literature. Corsino et al. (2021) [27] note that diversity and bias training initiatives are frequently implemented as isolated educational units rather than fully integrated into core curricula. They argue that EDIA efforts are more likely to have sustained impact when implemented institution wide and embedded withing learning and teaching structures rather than delivered as independent or add-on sessions (see also Louw et al. 2025 [28]). This isolated work on EDIA in pharmacy education suggests that comprehensive EDIA work at the program level is either not undertaken or poorly researched. It is likely that this isolated and fragmented work is the result of complex organizational and structural barriers to change, including study program design with little possibility for additional or altered curriculum, accreditation guidelines, and faculty workloads and fields of expertise. Additionally, most pharmacy educators are likely to receive limited professional acknowledgement for publications related to teaching. These barriers create a significant challenge for pharmacy educators, faculty developers and higher education researchers who are working to revise pharmacy courses and programs using a research-informed approach to teaching and curriculum development.

In addition to the isolated nature of EDIA research in pharmacy education, it is important to note fragmentation in how EDIA is approached. What typifies this research is a focus on single aspects of EDIA work (e.g., intercultural communication, heteronormativity in health care), which are often taken up through specific assignments or in special topics courses. It is less common that EDIA in pharmacy education is taken up through an intersectional approach. Crenshaw’s [29] concept of intersectionality was developed to theorize and examine how power relations (e.g., sexism, racism) work together and how our identities are multiple and constructed relationally (e.g., how one’s sexuality, disability and religion collectively shape identity). The concept of intersectionality has been used in research about students’ experiences in pharmacy and medical education [30,31], yet it is not well integrated into teaching strategies or curriculum work in pharmacy education.

By engaging with EDIA in pharmacy education through a more fragmented approach, there is also an imbalance in what areas of EDIA are attended to. For example, studies on intercultural competence or communication (ICC) are very common, while other areas are rarely addressed (e.g., migration/refugees/asylum seekers/physical and intellectual disability). A heavy focus on ICC competence and less focus on other aspects of EDIA may be due to its perceived on immediate patient–pharmacist relationships, patient safety and pharmacy care outcomes. It may also reflect national concerns, professional accreditation guidelines or institutional priorities, which may not be readily useful in other national or regional contexts. Moreover, the prolific focus on ICC is likely also tied to the ready availability of standardized survey tools for assessing ICC skills and values; this may be appealing to pharmacy educators who may gravitate to impacts that seem quantitatively measurable. Meanwhile, other aspects of EDIA may be complex to define, difficult to measure and challenging to implement through short-term teaching strategies or curriculum developments.

Significantly, many of the studies in this review emphasized that particular teaching strategies or curriculum efforts were highly dependent on the competence of just one or two staff members (e.g., curriculum on LGBTQ+ health developed by one staff member in a unit with expertise; development of a poverty simulation game dependent on a partnership between one staff member and a community organization). This trend in the literature suggests that EDIA work in pharmacy education is extremely vulnerable—should a certain staff member burn out, or leave a department, the resulting disappearance of EDIA work may be inevitable. If this is coupled with a lack of institutional support or prohibition of engaging in EDIA work (as is now experienced broadly in the USA), the continuity and development of existing work risks collapsing. Further still, it highlights the need for further faculty development opportunities in EDIA in pharmacy education to reduce the reliance on a small number of staff to carry this work.

The isolated and fragmented approach to EDIA in pharmacy education results in uneven engagement with aspects of EDIA and poses challenges for pharmacy programs. Most if not all pharmacy programs struggle to find ‘space’ for additional inclusions (new courses, new content, new teaching activities or strategies). Relying on a small number of educators to build up EDIA in pharmacy programs risks burnout for educators who are trying to build EDIA work into already packed programs. As such, approaches that are broadly integrated and intersectional in their approach are urgently needed if EDIA is to be strengthened in pharmacy education.

### 4.4. Research Objectives and Methods

The research included in this scoping review uses a very narrow range of research methods to investigate EDIA in pharmacy education. Specifically, this literature relies extremely heavily on the use of survey methods to assess nearly all possible research objectives. Except for review articles, surveys were used in most studies. The overreliance of survey research may show two key concerns for this area of research. First, the types of inquiries and research questions that can be asked and answered with survey research is limited. This is particularly relevant when considering that most research looked at singular educational contexts (research on one course or one teaching method with one group of students in a single year). Researchers repeatedly emphasize the limitation of the small scale or singular context studies—noting their limits in applicability to other contexts. Looking across this research though, the limitations may not be primarily about applicability but rather that it shapes the kinds of research questions asked and addressed. Second, we posit that the reliance on survey research in EDIA in pharmacy education research is likely related to who conducts the research. Most research is written primarily (23/86) or entirely (53/86) by scholars from pharmacy programs. For many pharmacy educators, surveys may bear the most similarity to research methods used in their home discipline, whereas arts-based approaches, interviews or other exclusively qualitative methods may be unfamiliar or perhaps perceived as less reliable or valid methods. Moreover, surveys (in particular, previously developed or validated) may be appealing for their efficiency of use, familiarity or perceived scholarly value. Thus, to expand and vary methods used in EDIA in pharmacy education research, it may be necessary to foster more interdisciplinary collaboration between pharmacy researchers and those from relevant and related disciplines (e.g., education, women and gender studies) who employ other methods in their research which may be unfamiliar, seem appear less scientifically valid or be perceived as more time consuming.

The objectives of EDIA in pharmacy education research are heavily clustered toward assessing the effectiveness of a practice or students’ comprehension of a particular topic area. Put simply, many of these studies query ‘did this work?’ or ‘do students remember?’ Relatively few studies investigated if or to what extent students developed a particular set of skills (6/83). Much of the EDIA research in pharmacy education is motivated by a stated need for the development of competence in EDIA in pharmaceutical care. This motivation may stem from changing requirements related to accreditation, guidelines from national organizations, institutional guidelines, and research on health inequalities. What drives much of this research on EDIA in pharmacy education is a clear need for competence among future pharmacists to provide inclusive health care. Yet, the objectives of the research included in this scoping review provide only partial views into whether students understand, adopt and become proficient in providing inclusive pharmaceutical care because of the teaching strategies and curriculum work. To better understand the impact of these educational initiatives, it will be necessary to engage in more varied and longer-term research projects that focus on implementation in practice. If we want to know not just if certain teaching practices or curriculum is effective, the research undertaken on EDIA in pharmacy education must be both more varied and more oriented toward how students engage with EDIA after their studies are completed.

It is, of course, demanding to engage in research that uses mixed methods, that looks at change over time and that grapples with the challenge of defining what counts as an implementation or adoption of practices when it comes to inclusive pharmaceutical care. But it is precisely this kind of work that this field most urgently needs. As educators both individually and collectively grapple with how to engage in educational change, the need for rich and varied research is amplified.

## 5. Limitations of the Study

This review has several limitations. While the search was not restricted by full-text language, inclusion required an abstract available in English, as this was necessary for initial screening. This may have led to the exclusion of relevant studies without English abstracts and may still contribute to the geographical concentration observed in the findings.

The evidence base was heavily dominated by studies from the USA, which may limit the applicability of findings to other educational and policy contexts. Moreover, EDIA work in pharmacy education in the USA, given the current political climate, has been significantly compromised. This review focused on published academic literature and did not include gray literature. As EDIA initiatives are often implemented and reported at the institutional level, relevant practices may not be captured in the published evidence base. A separate review of gray literature will provide a valuable supplement to this review.

EDIA work may occur through local teaching practices, curriculum development, institutional initiatives, or faculty development activities that are not published as research. Therefore, the limited representation of some EDIA topics in this review should not be interpreted as evidence that such work is absent in practice but rather that it is underrepresented in the scholarly literature. This also highlights the need to better support pharmacy educators in translating EDIA-related teaching, curriculum development and faculty development work into scholarly outputs.

Finally, the review focused on studies reporting implemented or evaluated EDIA initiatives within pharmacy education. Studies that examined preliminary curriculum exploration (e.g., needs assessments or curriculum mapping without implementation) were excluded. While this approach ensured alignment with the review objectives, it may limit insight into how EDIA initiatives are conceptualized and developed prior to implementation. The literature search was conducted in March 2025; therefore, more recent studies may not be included. The review process was conducted over an extended period, which may have contributed to the time between the final search and manuscript completion.

## 6. Conclusions and Future Directions

This scoping review shows that research on equity, diversity, inclusion, and accessibility (EDIA) in pharmacy education is expanding but remains uneven in scope and approach. The literature is heavily concentrated in the USA and largely focused on short-term, small-scale interventions, which are often delivered as standalone activities rather than embedded across curricula. Faculty development related to EDIA in pharmacy education, longitudinal integration, and intersectional approaches to EDIA remain underexplored.

These patterns are significant given the role of EDIA in shaping both student learning and future pharmacy practice. Inclusive learning environments can support students’ engagement, motivation, confidence, and sense of belonging, which are important for learning and professional development. At the same time, pharmacy education has a responsibility to prepare graduates to provide equitable and inclusive care to diverse patient populations. When EDIA is addressed in fragmented or isolated ways, this may limit both the educational impact and the development of competencies needed in practice.

Strengthening EDIA in pharmacy education requires greater and sustained support for faculty, as educators play a central role in shaping learning environments, curriculum, and professional norms. EDIA also needs to be embedded across programs rather than addressed through isolated modules or elective courses. This includes integrating EDIA principles across courses, assessments, and learning activities over time, which is supported by institutional commitment and resources.

Advancing EDIA in pharmacy education will require coordinated, program-level and institutional efforts that connect inclusive teaching, curriculum design, and professional preparation to better support both student learning and inclusive pharmaceutical care.

## Figures and Tables

**Table 1:** Definitions of EDIA terminology.

Term	Definition
equity	Equity describes the act of fairness and impartiality in the allocation of resources and opportunities. Unlike equality, which treats everyone the same, equity recognizes that individuals have different needs and seeks to offer tailored support to ensure everyone has an equal outcome [2].
diversity	Diversity refers to variety of societal identities and often emphasizes quantity. It includes differences in age, disability, gender, gender identity, ethnicity, race, religion, sexual orientation, ideas, values and responsibilities. Since people belong to multiple, evolving identity groups, their opportunities and outcomes can shift over time [2].
inclusion	Inclusion refers to how individuals and groups feel within a community or setting. It is achieved when people experience both a sense of belonging and feel recognized and valued as unique individuals, each with their own identity, experiences, and perspectives [2].
accessibility	Accessibility is defined as the provision of flexibility to accommodate an individual’s needs and preferences [3]. It ensures that all individuals, including those with disabilities, are able to exercise all rights and fundamental freedoms and are empowered to participate fully in equal terms with all others [4].

## Data Availability

No new data were created or analyzed in this study. Data sharing is not applicable to this article.

## Supplementary Materials

The following supporting information can be downloaded at https://www.mdpi.com/article/10.3390/pharmacy14030076/s1, File S1: PRISMA Checklist; File S2: Documentation of Searches; File S3: List of all studies; File S4: Data extraction tool. (References [32,33,34,35,36,37,38,39,40,41,42,43,44,45,46,47,48,49,50,51,52,53,54,55,56,57,58,59,60,61,62,63,64,65,66,67,68,69,70,71,72,73,74,75,76,77,78,79,80,81,82,83,84,85,86,87,88,89,90,91,92,93,94,95,96,97,98,99,100,101,102,103,104,105,106,107,108,109,110,111,112,113,114] are cited in the supplementary materials).

## Abbreviations

The following abbreviations are used in this manuscript:

EDIA	Equity, Diversity, Inclusion, and Accessibility
JBI	Joanna Briggs Institute
PCC	Population, Concept, and Context
SDOH	Social Determinants
LGBQ+	Lesbian Gay Bisexual Queer plus
