# Supplementary material for: Equity, Diversity, Inclusion and Accessibility in Pharmacy Education: A Scoping Review
Source: Pharmacy (Basel). 2026 May 20;14(3):76. doi: 10.3390/pharmacy14030076 (PMC13214706; doi:10.3390/pharmacy14030076)
Supplement: Supplementary file 1 [file pharmacy-14-00076-s001.zip › File S2 Documentation of Searches.pdf]

Supplementary Material

Documentation of searches in Embase, Medline, ERIC, CINAHL, Web of Science and APA PsycINFO; Search date 25 March 2025

Embase (Ovid)<1974 to 2025 March 24>; Search date 25 March 2025

1 pharmacy education/ or pharmacy school/ 3914
2 pharmacy student/ 10650
3 (pharmac* adj3 (student* or learner* or educat* or curricul* or school* or residenc* or facult* or teach* or instruct* or professor* or staff or train* or practic* or placement or counseling)).ti,ab,kf. 51849
4 curriculum/ or curriculum development/ 119932
5 academia/ or continuing education/ or continuing education provider/ or education program/ or educational technology/ or interprofessional education/ or interdisciplinary education/ or learning environment/ or lifelong learning/ or mentoring/ 112739
6 ((faculty or education* or curriculum or instruction* or program or teaching) adj3 (development or planning or technology)).ti,ab,kf. 54577
7 ((education* or learning or teaching) adj2 (continuing or program or interprofessional or inter-professional or interdisciplinary or multiprofessional or multi-professional or multidisciplinary or environment or lifelong)).ti,ab,kf. 94312
8 (mentor* or "professionalism in practice").ti,ab,kf. 34488
9 ((teaching or education*) adj3 (method* or strateg*)).ti,ab,kf. 60213
10 or/4-9 380565
11 pharmacology/ or pharmac*.ti,kf. 622062
12 10 and 11 13130
13 1 or 2 or 3 or 12 58367
14 inclusive education/ 497
15 social justice/ or antiracism/ or social inclusion/ or social inequality/ or social exclusion/ 20541
16 cultural diversity/ or "diversity, equity and inclusion"/ 5877
17 cultural competence/ or multilingualism/ 11714
18 colonialism/ or empowerment/ or intersectionality/ 18338
19 color discrimination/ or auditory discrimination/ or social discrimination/ or colorism/ or disability discrimination/ or "discrimination against sexual and gender minorities"/ or racism/ or homophobia/ or xenophobia/ or sexism/ 37514
20 special education/ or "education of intellectually disabled"/ or "education of visually disabled"/ or disabled student/ 4638
21 "ethnic or racial aspects"/ or ethnicity/ or race/ 232555
22 population group/ or ethnic group/ or minority group/ or religious group/ 108144
23 migration/ or immigration/ or immigrant/ or undocumented immigrant/ or refugee/87263
24 "sexual and gender minority"/ or exp lgbtqia+ people/ or sexuality/ or transsexuality/ or sexual orientation/ or bisexuality/ or heterosexuality/ or homosexuality/ 123868
25 social class/ or socioeconomics/ or lowest income group/ 238467
26 (mental health/ or psychological well-being/) and (student* or learner* or educat* or curricul* or school* or residenc* or facult* or teach* or instruct* or professor* or staff or train*).ti,ab,kf. 75645
27 religion/ 79860

28 (EDIA or EDI or equ* divers* inclus* or DEI or DEIA or DEAI or DEIB or divers* equ* inclus* or JEDI or EDIJ).ti,ab,kf. 9386
29 ((equity or equality or inequity or inequality) adj3 (divers* or inclus* or educat* or learning or accessib* or pharmac*)).ti,ab,kf. 6972
30 (inclusiveness or inclusivity or ((inclusion or inclusive or equity or exclusion) adj3 (education or learning or teaching or classroom or pharmac*))).ti,ab,kf. 10131
31 ("Access to Education" or (Accessib* adj3 disab*) or "universal design for learning" or UDL).ti,ab,kf. 2258
32 ((intercultural* or inter-cultural* or crosscultural* or cross-cultural* or multicultural* or multi-cultural* or transcultural* or trans-cultural*) adj3 (communication or training or program* or education)).ti,ab,kf. 1745
33 ((cultur* or cross-cultur*) adj2 (awareness or difference* or pluralism or foreign or conflict* or sensitivity or responsive* or divers* or humility or competenc* or safety)).ti,ab,kf. 45406
34 (multilingual material* or multilingualism).ti,ab,kf. 440
35 (colonialism or decoloni?ation or empowerment or intersectionality or postcolonialism).ti,ab,kf. 32860
36 ((first generation or special needs) adj3 student*).ti,ab,kf. 620
37 ((disability* or handicap* or able-ism or ableism or educational or gender or racial) adj3 (discrimination or attitude* or student* or difference*)).ti,ab,kf. 125118
38 (race or racism or antiracism or ethnicity or ((ethnic or minority or racial) adj3 (diversity or group* or attitude*))).ti,ab,kf. 408484
39 (immigration or immigrant* or migrant* or refugee* or paperless).ti,ab,kf. 87154
40 (LGBT* or homosexual* or sexuality or bisexual* or heterosexual* or transsexual* or transgender*).ti,ab,kf. 95329
41 (social class or socioeconomic status or low income student* or social justice or social inclusion or social marginali* or social exclusion or social rejection).ti,ab,kf. 98984
42 (neurodivergen* or neurodivers* or ((mental health or wellbeing or well-being) adj3 (student* or learner* or faculty or teacher* or educator* or instructor* or professor* or staff or train*))).ti,ab,kf. 17675
43 (Religio* or Buddhis* or Christian* or Confucian* or Islam* or Judaism or Hindu* or anthroposoph* or Mormon* or shaman* or spiritualism or taoism or daoism or voodoo or atheis*).ti,ab,kf. 86309
44 or/14-43 1421045
45 13 and 44 4060
46 limit 45 to conference abstract 1145
47 45 not 46 2915
48 limit 47 to yr="2014 -Current" 2151

https://ovidsp.ovid.com/ovidweb.cgi?T=JS&NEWS=N&PAGE=main&SHAREDSEARCHID=1v1bbDjUJvT8ix6mJUhVr4qNlziJRBlAtiZ42KNJ1EnJ2P7ZZ4mNd6J4KOqAhulG

Comments to OVID-databases:

- / = search on subject heading
- Exp = search on a subject heading, expanded to include narrower terms
- Ti,ab,kf = search in title, abstract and authors keywords of the article
- Adjn = The adjacency operator
  (ADJn) retrieves records that contain search terms within a specified number (*n-1*) of words from each other in any order

Ovid MEDLINE(R) and Epub Ahead of Print, In-Process, In-Data-Review & Other Non-Indexed Citations, Daily and Versions <1946 to March 24, 2025>; Search date 25 March 2025

1 education, pharmacy/ or education, pharmacy, continuing/ or education, pharmacy, graduate/ or pharmacy residencies/ 10174
2 Students, Pharmacy/ or Schools, Pharmacy/ or Faculty, Pharmacy/ 6144
3 (pharmac* adj3 (student* or learner* or educat* or curricul* or school* or residenc* or facult* or teach* or instruct* or professor* or staff or train* or practic* or placement or counseling)).ti,ab,kf. 29322
4 curriculum/ or interdisciplinary studies/ 93977
5 academia/ or education, professional/ or interdisciplinary placement/ or Educational Technology/ or Program Development/ or mentors/ 49069
6 ((faculty or education* or curriculum or instruction* or program or teaching) adj3 (development or planning or technology)).ti,ab,kf. 46377
7 ((education* or learning or teaching) adj2 (continuing or program or interprofessional or inter-professional or interdisciplinary or multiprofessional or multi-professional or multidisciplinary or environment or lifelong)).ti,ab,kf. 73123
8 (mentor* or "professionalism in practice").ti,ab,kf. 26701
9 ((teaching or education*) adj3 (method* or strateg*)).ti,ab,kf. 36077
10 4 or 5 or 6 or 7 or 8 or 9 274196
11 pharmacology/ or pharmac*.ti,kf. 424420
12 10 and 11 6654
13 1 or 2 or 3 or 12 34716
14 Social Justice/ or Antiracism/ or social inclusion/ or social marginalization/ 15891
15 cultural diversity/ or diversity, equity, inclusion/ or workforce diversity/ 14228
16 Cultural Competency/ or multilingualism/ 13509
17 Colonialism/ or empowerment/ or intersectional framework/ 3432
18 bias, implicit/ or disability discrimination/ or homophobia/ or racism/ or systemic racism/ or sexism/ or xenophobia/ 12818
19 education, special/ or "education of hearing disabled"/ or "education of intellectually disabled"/ or "education of visually disabled"/ 15147
20 ethnicity/ or racial groups/ 97667
21 "ethnic and racial minorities"/ or minority groups/ 19923
22 "Emigration and Immigration"/ or "emigrants and immigrants"/ or undocumented immigrants/ or Refugees/ 54148
23 sexuality/ or bisexuality/ or heterosexuality/ or exp homosexuality/ or transsexualism/ 52441
24 "sexual and gender minorities"/ or gender-nonconforming persons/ or intersex persons/ or transgender persons/ or gender equity/ 22198
25 social class/ or low socioeconomic status/ 46510
26 mental health/ and (student* or learner* or educat* or curricul* or school* or residenc* or facult* or teach* or instruct* or professor* or staff or train*).ti,ab,kf. 20028
27 religion/ 16275
28 (EDIA or EDI or equ* divers* inclus* or DEI or DEIA or DEAI or DEIB or divers* equ* inclus* or JEDI or EDIJ).ti,ab,kf. 9005
29 ((equity or equality or inequity or inequality) adj3 (divers* or inclus* or educat* or learning or accessib* or pharmac*)).ti,ab,kf. 5878

30 (inclusiveness or inclusivity or ((inclusion or inclusive or equity or exclusion) adj3 (education or learning or teaching or classroom or pharmac*))).ti,ab,kf. 7620
31 ("Access to Education" or (Accessib* adj3 disab*) or "universal design for learning" or UDL).ti,ab,kf. 1770
32 ((intercultural* or inter-cultural* or crosscultural* or cross-cultural* or multicultural* or multi-cultural* or transcultural* or trans-cultural*) adj3 (communication or training or program* or education)).ti,ab,kf. 1511
33 ((cultur* or cross-cultur*) adj2 (awareness or difference* or pluralism or foreign or conflict* or sensitivity or responsive* or divers* or humility or competenc* or safety)).ti,ab,kf. 36856
34 (multilingual material* or multilingualism).ti,ab,kf. 449
35 (colonialism or decoloni?ation or empowerment or intersectionality or postcolonialism).ti,ab,kf. 27693
36 ((first generation or special needs) adj3 student*).ti,ab,kf. 536
37 ((disability* or handicap* or able-ism or ableism or educational or gender or racial) adj3 (discrimination or attitude* or student* or difference*)).ti,ab,kf. 90057
38 (race or racism or antiracism or ethnicity or ((ethnic or minority or racial) adj3 (diversity or group* or attitude*))).ti,ab,kf. 273496
39 (immigration or immigrant* or migrant* or refugee* or paperless).ti,ab,kf. 79250
40 (LGBT* or homosexual* or sexuality or bisexual* or heterosexual* or transsexual* or transgender*).ti,ab,kf. 76074
41 (social class or socioeconomic status or low income student* or social justice or social inclusion or social marginali* or social exclusion or social rejection).ti,ab,kf. 83954
42 (neurodivergen* or neurodivers* or ((mental health or wellbeing or well-being) adj3 (student* or learner* or faculty or teacher* or educator* or instructor* or professor* or staff or train*))).ti,ab,kf. 15739
43 (Religio* or Buddhis* or Christian* or Confucian* or Islam* or Judaism or Hindu* or anthroposoph* or Mormon* or shaman* or spiritualism or taoism or daoism or voodoo or atheis*).ti,ab,kf. 74276
44 or/14-43 879482
45 13 and 44 1945
46 limit 45 to yr="2014 -Current" 1485

https://ovidsp.ovid.com/ovidweb.cgi?T=JS&NEWS=N&PAGE=main&SHAREDSEARCHID=29fWO146jyezEfxXud5Ky17dhZMTkNkyGIXVq4gMYggDzhUJy5pkBNPxRgOiJpnle

ERIC Education Resource Information Center (Ebsco); 1966 to current; Search date 25 March 2025

S1 DE "Pharmacy" OR DE "Pharmacology" OR TI pharmac* OR AB pharmac* 4,567
S2 (DE "Faculty Development") OR (DE "Educational Development" OR DE "Curriculum Development" OR DE "Instructional Development" OR DE "Educational Planning" OR DE "Educational Methods" OR DE "Educational Strategies" OR DE "Educational Research" OR DE "Educational Technology" OR DE "Program Development" OR DE "Mentors" OR DE "Teaching Methods") 477,570
S3 S1 AND S2 830

S4 TI (pharmac* N2 (student* or learner* or educat* or curricul* or school* or residenc* or facult* or teach* or instruct* or professor* or staff or train* or practic* or placement or counseling)) OR AB (pharmac* N2 (student* or learner* or educat* or curricul* or school* or residenc* or facult* or teach* or instruct* or professor* or staff or train* or practic* or placement or counseling)) 1,484

S5 TI ((faculty or education* or curriculum or instruction* or teaching or program) N2 (development or planning or technology)) OR AB ((faculty or education* or curriculum or instruction* or teaching or or program) N2 (development or planning or technology)) 100,672

S6 TI ((education* or learning or teaching) N1 (continuing or program or interprofessional or inter-professional or interdisciplinary or multiprofessional or multi-professional or multidisciplinary or environment or lifelong)) OR AB ((education* or learning or teaching) N1 (continuing or program or interprofessional or inter-professional or interdisciplinary or multiprofessional or multi-professional or multidisciplinary or environment or lifelong)) 149,978

S7 TI (((mentor* or "professionalism in practice" or ((teaching or education*) N2 (method* or strateg*))) OR AB (((mentor* or "professionalism in practice") or ((teaching or education*) N2 (method* or strateg*)) 295,975

S8 S5 OR S6 OR S7 481,452

S9 S1 AND S8 991

S10 S3 OR S4 OR S9 2,152

S11 DE "Inclusion" OR DE "Equal Education" OR DE "Justice" 51,865

S12 DE "Diversity (Faculty)" OR DE "Student Diversity" 12,605

S13 DE "Access to Education" OR DE "Accessibility (for Disabled)" 31,560

S14 DE "Intercultural Communication" OR DE "Communication Problems" OR DE "Cross Cultural Training" OR DE "Cultural Awareness" OR DE "Cultural Differences" OR DE "Cultural Pluralism" OR DE "Foreign Culture" OR DE "Intercultural Programs" OR DE "Multicultural Education" OR DE "Multilingual Materials" OR DE "Multilingualism" 76,491

S15 DE "Colonialism" OR DE "Decolonization" OR DE "Empowerment" OR DE "Intersectionality" OR DE "Postcolonialism" 8,121

S16 DE "First Generation College Students" 3,009

S17 DE "Educational Discrimination" OR DE "Gender Discrimination" OR DE "Gender Differences" 49,786

S18 DE "Disability Discrimination" OR DE "Attitudes toward Disabilities" OR DE "Students with Disabilities" OR DE "Special Needs Students" 25,025

S19 DE "Race" OR DE "Ethnicity" OR DE "Culture Conflict" OR DE "Ethnic Diversity" OR DE "Ethnic Groups" OR DE "Minority Groups" OR DE "Racial Attitudes" OR DE "Racism" OR DE "Racial Discrimination" OR DE "Minority Group Students" 79,859

S20 DE "Immigration" OR DE "Immigrants" OR DE "Undocumented Immigrants" OR DE "Migrant Education" OR DE "Migrants" OR DE "Immigrants" OR DE "Migrant Children" OR DE "Migrant Workers" OR DE "Refugees" OR DE "Refugees" OR DE "Undocumented Immigrants" 25,706

S21 DE "LGBTQ People" OR DE "Sexual Orientation" OR DE "Homosexuality" 7,862

S22 DE "Social Class" OR DE "Socioeconomic Status" OR DE "Low Income Students" 27,276

S23 DE "Mental Health" 18,462

S24 TI (student* or learner* or educat* or curricul* or school* or residenc* or facult* or teach* or instruct* or professor* or staff or train*) OR AB (student* or learner* or educat* or curricul* or school* or residenc* or facult* or teach* or instruct* or professor* or staff or train*) 1,603,151

S25 S23 AND S24 12,723
S26 DE "Religion" OR DE "Religious Cultural Groups" 9,263
S27 TI (EDIA or EDI or equ* divers* inclus* or DEI or DEIA or DEAI or DEIB or divers* equ* inclus* or JEDI or EDIJ) OR AB (EDIA or EDI or equ* divers* inclus* or DEI or DEIA or DEAI or DEIB or divers* equ* inclus* or JEDI or EDIJ) 1,491
S28 TI ((equity or equality or inequity or inequality) N2 (divers* or inclus* or educat* or learning or accessib* or pharmac*)) OR AB ((equity or equality or inequity or inequality) N2 (divers* or inclus* or educat* or learning or accessib* or pharmac*)) 13,322
S29 TI (inclusiveness or inclusivity or ((inclusion or inclusive or equity or exclusion) N2 (education or learning or teaching or classroom or pharmac*))) OR AB (inclusiveness or inclusivity or ((inclusion or inclusive or equity or exclusion) N2 (education or learning or teaching or classroom or pharmac*))) 16,102
S30 TI ("Access to Education" or (Accessib* N2 disab*) or "universal design for learning" or UDL) OR AB ("Access to Education" or (Accessib* N2 disab*) or "universal design for learning" or UDL) 3,065
S31 TI ((intercultural* or inter-cultural* or crosscultural* or cross-cultural* or multicultural* or multi-cultural* or transcultural* or trans-cultural*) N2 (communication or training or program* or education)) OR AB ((intercultural* or inter-cultural* or crosscultural* or cross-cultural* or multicultural* or multi-cultural* or transcultural* or trans-cultural*) N2 (communication or training or program* or education)) 10,001
S32 TI ((cultur* or cross-cultur*) N1 (awareness or difference* or pluralism or foreign or conflict* or sensitivity or responsive* or divers* or humility or competenc* or safety)) OR AB ((cultur* or cross-cultur*) N1 (awareness or difference* or pluralism or foreign or conflict* or sensitivity or responsive* or divers* or humility or competenc* or safety)) 23,479
S33 TI ("multilingual material*" or multilingualism) OR AB ("multilingual material*" or multilingualism) 1,723
S34 TI (colonialism or decoloni#ation or empowerment or intersectionality or postcolonialism) OR AB (colonialism or decoloni#ation or empowerment or intersectionality or postcolonialism) 11,266
S35 TI (("first generation" or "special needs") N2 student*) OR AB (("first generation" or "special needs") N2 student*) 6,479
S36 TI ((disability or handicap* or able-ism or ableism or educational or gender or racial) N2 (discrimination or attitude* or student* or difference*)) OR AB ((disability or handicap* or able-ism or ableism or educational or gender or racial) N2 (discrimination or attitude* or student* or difference*)) 59,764
S37 TI (race or racism or antiracism or ethnicity or ((ethnic or minority or racial) N2 (diversity or group* or attitude*))) OR AB (race or racism or antiracism or ethnicity or ((ethnic or minority or racial) N2 (diversity or group* or attitude*))) 63,390
S38 TI (immigration or immigrant* or migrant* or refugee* or paperless) OR AB (immigration or immigrant* or migrant* or refugee* or paperless) 25,027
S39 TI (LGBT* or homosexual* or sexuality or bisexual* or heterosexual* or transsexual* or transgender*) OR AB (LGBT* or homosexual* or sexuality or bisexual* or heterosexual* or transsexual* or transgender*) 10,088
S40 TI ("social class" or "socioeconomic status" or "low income student*" or "social justice" or "social inclusion" or "social marginali*" or "social exclusion" or "social rejection") OR AB ("social class" or "socioeconomic status" or "low income student*" or "social justice" or "social inclusion" or "social marginali*" or "social exclusion" or "social rejection") 28,406
S41 TI (neurodivergen* or neurodivers* or ((mental health or wellbeing or well-being) N2 (student* or learner* or faculty or teacher* or educator* or instructor* or professor* or staff or

train*))) OR AB (neurodivergen* or neurodivers* or ((mental health or wellbeing or well-being) N2 (student* or learner* or faculty or teacher* or educator* or instructor* or professor* or staff or train*))) 7,503
S42 TI (Religio* or Buddhis* or Christian* or Confucian* or Islam* or Judaism or Hindu* or anthroposoph* or Mormon* or shaman* or spiritualism or taoism or daoism or voodoo or atheis*) OR AB (Religio* or Buddhis* or Christian* or Confucian* or Islam* or Judaism or Hindu* or anthroposoph* or Mormon* or shaman* or spiritualism or taoism or daoism or voodoo or atheis*) 28,929
S43 S11 OR S12 OR S13 OR S14 OR S15 OR S16 OR S17 OR S18 OR S19 OR S20 OR S21 OR S22 OR S25 OR S26 OR S27 OR S28 OR S29 OR S30 OR S31 OR S32 OR S33 OR S34 OR S35 OR S36 OR S37 OR S38 OR S39 OR S40 OR S41 OR 42 408,214
S44 S10 AND S43 201
S45 S44 (Limiters - Published Date: 20140101-20251231) 67

Comments on Ebsco databases:

- TI,AB = Title, Abstract
- XB = search in title and abstract (for CINAHL)
- DE = Descriptor (ERIC)
- MH = medical subject heading in CINAHL
- Nn = The adjacency operator (N) retrieves records that contain search terms within a specified number (n) of words from each other in any order

CINAHL (Ebsco) 1981 -current; Search date 25 March 2025

S1 (MH "Education, Pharmacy") 2,176
S2 (MH "Students, Pharmacy") 1,697
S3 XB (pharmac* N2 (student* or learner* or educat* or curricul* or school* or residenc* or facult* or teach* or instruct* or professor* or staff or train* or practic* or placement or counseling)) 10,384
S4 (MH "Curriculum Development") OR (MH "Curriculum") OR (MH "Integrated Curriculum") 41,599
S5 (MH "Education, Continuing") OR (MH "Education, Interdisciplinary") 21,695
S6 (MH "Academia") OR (MH "Faculty Development") OR (MH "Program Development") OR (MH "Program Planning") OR (MH "Learning Environment") OR (MH "Educational Technology") OR (MH "Teaching Methods") OR (MH "Mentorship") 105,558
S7 XB ((faculty or education* or curriculum or instruction* or teaching or program) N2 (development or planning or technology)) 28,610
S8 XB ((education* or learning or teaching) N1 (continuing or program or interprofessional or inter-professional or interdisciplinary or multiprofessional or multi-professional or multidisciplinary or environment or lifelong)) 67,852
S9 XB (((mentor* or "professionalism in practice" or ((teaching or education*) N2 (method* or strateg*))) 40,411
S10 S4 OR S5 OR S6 OR S7 OR S8 OR S9 240,998
S11 (MH "Pharmacy and Pharmacology") OR TI pharmac* OR AB pharmac* 170,949
S12 S10 AND S11 5,187
S13 S1 OR S2 OR S3 OR S12 15,075
S14 (MH "Social Justice") OR (MH "Antiracism") OR (MH "Social Inclusion") 17,090

S15 (MH "Cultural Diversity") OR (MH "Diversity, Equity, Inclusion") OR (MH "Gender Neutrality") OR (MH "Workforce Diversity") 20,586
S16 (MH "Cultural Competence") OR (MH "Multilingualism") 17,880
S17 (MH "Empowerment") OR (MH "Intersectionality") OR (MH "Decolonization") 20,945
S18 (MH "Implicit Bias") OR (MH "Disability Discrimination") OR (MH "Racism") OR (MH "Xenophobia") OR (MH "Transphobia") OR (MH "Homophobia") OR (MH "Sexism") OR (MH "Systemic Racism") OR (MH "Persons with Disabilities") OR (MH "Students with Disabilities") OR (MH "Persons with Visual Disabilities") OR (MH "Persons with Hearing Disabilities") 65,804
S19 (MH "Education, Special") 7,563
S20 (MH "Ethnic Groups") OR (MH "Racial Equality") OR (MH "Minority Groups") OR (MH "Racialization") 49,511
S21 (MH "Emigration and Immigration") OR (MH "Undocumented Immigrants") OR (MH "Immigrants") OR (MH "Migrants") OR (MH "Refugees") 38,935
S22 (MH "Sexuality") OR (MH "Heterosexuality") OR (MH "Bisexuality") OR (MH "Sexual Orientation") OR (MH "Homosexuality") 42,433
S23 (MH "LGBTQ+ Persons") OR (MH "Gender-Nonconforming Persons") OR (MH "Intersex Persons") OR (MH "Sexual and Gender Minorities") OR (MH "Transgender Persons") OR (MH "Gender Equality") 15,453
S24 (MH "Social Class") OR (MH "Low Socioeconomic Status") 16,763
S25 (MH "Mental Health") 68,286
S26 XB (student* or learner* or educat* or curricul* or school* or residenc* or facult* or teach* or instruct* or professor* or staff or train*) 1,077,206
S27 S25 AND S26 16,935
S28 (MH "Religion and Religions")15,460
S29 XB (EDIA or EDI or equ* divers* inclus* or DEI or DEIA or DEAI or DEIB or divers* equ* inclus* or JEDI or EDIJ) 4,296
S30 XB ((equity or equality or inequity or inequality) N2 (divers* or inclus* or educat* or learning or accessib* or pharmac*)) 3,685
S31 XB (inclusiveness or inclusivity or ((inclusion or inclusive or equity or exclusion) N2 (education or learning or teaching or classroom or pharmac*))) 4,371
S32 XB ("Access to Education" or (Accessib* N2 disab*) or "universal design for learning" or UDL) 832
S33 XB ((intercultural* or inter-cultural* or crosscultural* or cross-cultural* or multicultural* or multi-cultural* or transcultural* or trans-cultural*) N2 (communication or training or program* or education)) 1,269
S34 XB ((cultur* or cross-cultur*) N1 (awareness or difference* or pluralism or foreign or conflict* or sensitivity or responsive* or divers* or humility or competenc* or safety)) 21,791
S35 XB ("multilingual material*" or multilingualism) 136
S36 XB (colonialism or decoloni#ation or empowerment or intersectionality or postcolonialism) 18,169
S37 XB (("first generation" or "special needs") N2 student*) 380
S38 XB ((disability or handicap* or able-ism or ableism or educational or gender or racial) N2 (discrimination or attitude* or student* or difference*)) 40,768
S39 XB (race or racism or antiracism or ethnicity or ((ethnic or minority or racial) N2 (diversity or group* or attitude*))) 105,933
S40 XB (immigration or immigrant* or migrant* or refugee* or paperless) 35,224

S41 XB (LGBT* or homosexual* or sexuality or bisexual* or heterosexual* or transsexual* or transgender*) 35,709
S42 XB ("social class" or "socioeconomic status" or "low income student*" or "social justice" or "social inclusion" or "social marginali*" or "social exclusion" or "social rejection") 33,941
S43 (MH "Neurodiversity") OR XB (neurodivergen* or neurodivers* or ((mental health or wellbeing or well-being) N2 (student* or learner* or faculty or teacher* or educator* or instructor* or professor* or staff or train*))) 9,539
S44 XB (Religio* or Buddhis* or Christian* or Confucian* or Islam* or Judaism or Hindu* or anthroposoph* or Mormon* or shaman* or spiritualism or taoism or daoism or voodoo or atheis*) 33,933
S45 S14 OR S15 OR S16 OR S17 OR S18 OR S19 OR S20 OR S21 OR S22 OR S23 OR S24 OR S27 OR S28 OR S29 OR S30 OR S31 OR S32 OR S33 OR S34 OR S35 OR S36 OR S37 OR S38 OR S39 OR S40 OR S41 OR S42 OR S43 OR S44 486,393
S46 S13 AND S45 891
S47 S46 (Limiters - Publication Date: 20140101) 684

Web of Science (Clarivate), covering WOS.SCI: 1945 to 2025, WOS.AHCI: 1975 to 2025, WOS.ESCI: 2020 to 2025, WOS.SSCI: 1956 to 2025; Search date 25 March 2025
Searches are done as "exact searches"

1: TS=(((faculty or education* or curriculum or instruction* or program or teaching) NEAR/2 (development or planning or technology))) Results: 92143
2: TS=(((education* or learning or teaching) NEAR/1 (continuing or program or interprofessional or inter-professional or interdisciplinary or multiprofessional or multi-professional or multidisciplinary or environment or lifelong))) Results: 104083
3: TS=((mentor* or "professionalism in practice" or ((teaching or education*) NEAR/2 (method* or strateg*)))) Results: 107618
4: #1 OR #2 OR #3 Results: 281100
5: TS=(pharmac*) Results: 1254188
6: #4 AND #5 Results: 7851
7: TS=((pharmac* NEAR/2 (student* or learner* or educat* or curricul* or school* or residenc* or facult* or teach* or instruct* or professor* or staff or train* or practic* or placement or counseling))) Results: 28790
8: #6 OR #7 Results: 33040
9: TS=((EDIA or EDI or "equ* divers* inclus*" or DEI or DEIA or DEAI or DEIB or "divers* equ* inclus*" or JEDI or EDIJ)) Results: 14640
10: TS=(((equity or equality or inequity or inequality) NEAR/2 (divers* or inclus* or educat* or learning or accessib* or pharmac*))) Results: 18073
11: TS=((inclusiveness or inclusivity or ((inclusion or inclusive or equity or exclusion) NEAR/2 (education or learning or teaching or classroom or pharmac*)))) Results: 26392
12: TS=(("Access to Education" or (Accessib* NEAR/2 disab*) or "universal design for learning" or UDL)) Results: 3717
13: TS=(((intercultural* or inter-cultural* or crosscultural* or cross-cultural* or multicultural* or multi-cultural* or transcultural* or trans-cultural*) NEAR/2 (communication or training or program* or education))) Results: 9544

14: TS=(((cultur* or cross-cultur*) NEAR/1 (awareness or difference* or pluralism or foreign or conflict* or sensitivity or responsive* or divers* or humility or competenc* or safety))) Results: 72923

15: TS=(("multilingual material*" or multilingualism)) Results: 5938

16: TS=((colonialism or decoloni$ation or empowerment or intersectionality or postcolonialism)) Results: 96936

17: TS=((("first generation" or "special needs") NEAR/2 student*)) Results: 2205

18: TS=(((disability* or handicap* or able-ism or ableism or educational or gender or racial) NEAR/2 (discrimination or attitude* or student* or difference*))) Results: 200637

19: TS=((race or racism or antiracism or ethnicity or ((ethnic or minority or racial) NEAR/2 (diversity or group* or attitude*)))) Results: 457746

20: TS=((immigration or immigrant* or migrant* or refugee* or paperless)) Results: 219763

21: TS=((LGBT* or homosexual* or sexuality or bisexual* or heterosexual* or transsexual* or transgender*)) Results: 138512

22: TS=(("social class" or "socioeconomic status" or "low income student*" or "social justice" or "social inclusion" or "social marginali*" or "social exclusion" or "social rejection")) Results: 152276

23: TS=((neurodivergen* or neurodivers* or (("mental health" or wellbeing or well-being) NEAR/2 (student* or learner* or faculty or teacher* or educator* or instructor* or professor* or staff or train*)))) Results: 23791

24: TS=((Religio* or Buddhis* or Christian* or Confucian* or Islam* or Judaism or Hindu* or anthroposoph* or Mormon* or shaman* or spiritualism or taoism or daoism or voodoo or atheis*)) Results: 420981

25: #9 OR #10 OR #11 OR #12 OR #13 OR #14 OR #15 OR #16 OR #17 OR #18 OR #19 OR #20 OR #21 OR #22 OR #23 OR #24 Results: 1644470

26: #8 AND #25 Timespan: 2014-01-01 to 2025-12-31 Results: 1507

Comments:

- TS or (Topic) = Topic search = search in title, abstract and author key words
- Near/2 = The adjacency operator (NEAR/**n**) retrieves records that contain search terms within a specified number (*n*) of words between the search terms in any order

https://www.webofscience.com/wos/woscc/summary/a9bf4dae-dfe8-474e-9958-84ceea62c18c-015542682b/relevance/1

APA PsycInfo <1806 to March 2025 Week 3>; Search date 25 March 2025

1 (pharmac* adj3 (student* or learner* or educat* or curricul* or school* or residenc* or facult* or teach* or instruct* or professor* or staff or train* or practic* or placement or counseling)).tw. 3412

2 curriculum/ or curriculum development/ 38565

3 educational personnel/ or educational programs/ or mentor/ or teaching methods/ 100283

4 ((faculty or education* or curriculum or instruction* or program or teaching) adj3 (development or planning or technology)).tw. 51201

5 ((education* or learning or teaching) adj2 (continuing or program or interprofessional or inter-professional or interdisciplinary or multiprofessional or multi-professional or multidisciplinary or environment or lifelong)).tw. 52447
6 (mentor* or "professionalism in practice").tw. 23676
7 ((teaching or education*) adj3 (method* or strateg*)).tw. 36204
8 2 or 3 or 4 or 5 or 6 or 7 238190
9 pharmacology/ or pharmac*.tw. 105988
10 8 and 9 1587
11 1 or 10 4453
12 inclusive education/ or equal education/ or educational access/ 4183
13 social justice/ or antiracism/ or "racial and ethnic attitudes"/ or social inclusion/ or social equity/ or social exclusion/ or social equality/ or gender equality/ or gender gap/ 34597
14 diversity/ or "equity, diversity, and inclusion"/ or cultural diversity/ or diversity in the workplace/ or ethnic diversity/ or gender diversity/ or racial diversity/ 20682
15 equity/ or inclusion/ or disability inclusion/ or gender inclusion/ or organizational inclusion/ 7311
16 cultural competence/ or multilingualism/ or cross cultural differences/ 62597
17 colonialism/ or decolonization/ or empowerment/ or intersectionality/ 16573
18 social discrimination/ or disability discrimination/ or "race and ethnic discrimination"/ or sex discrimination/ or social class bias/ or Auditory Discrimination/ or Visual Discrimination/ or "Race and Ethnic Discrimination"/ 29186
19 racism/ or implicit bias/ or racial bias/ or sexual attitudes/ or "homosexuality (attitudes toward)"/ or "transgender (attitudes toward)"/ or stranger reactions/ 26775
20 special education/ or special education students/ 36074
21 ethnic identity/ or ethnic bias/ or ethnic diversity/ or multiracial/ or "racial and ethnic attitudes"/ or "racial and ethnic groups"/ or minority groups/ or sexual minority groups/ or religious groups/ or marginalized groups/ 64865
22 immigration/ or human migration/ or undocumented immigration/ or "immigration (attitudes toward)"/ or refugees/ 46076
23 sexual minority groups/ or sexual orientation/ or asexuality/ or heterosexuality/ or exp lgbtq/ or pansexuality/55681
24 social class/ or disadvantaged/ or lower socioeconomic status/ 18919
25 (neurodiversity/ or mental health/ or well being/) and (student* or learner* or educat* or curricul* or school* or residenc* or facult* or teach* or instruct* or professor* or staff or train*).tw. 55808
26 religion/ 22553
27 (EDIA or EDI or equ* divers* inclus* or DEI or DEIA or DEAI or DEIB or divers* equ* inclus* or JEDI or EDIJ).tw. 4130
28 ((equity or equality or inequity or inequality) adj3 (divers* or inclus* or educat* or learning or accessib* or pharmac*)).tw. 6369
29 (inclusiveness or inclusivity or ((inclusion or inclusive or equity or exclusion) adj3 (education or learning or teaching or classroom or pharmac*))).tw. 11585
30 ("Access to Education" or (Accessib* adj3 disab*) or "universal design for learning" or UDL).tw. 1774
31 ((intercultural* or inter-cultural* or crosscultural* or cross-cultural* or multicultural* or multi-cultural* or transcultural* or trans-cultural*) adj3 (communication or training or program* or education)).tw. 6479
32 ((cultur* or cross-cultur*) adj2 (awareness or difference* or pluralism or foreign or conflict* or sensitivity or responsive* or divers* or humility or competenc* or safety)).tw. 54401

33 (multilingual material* or multilingualism).tw. 1727
34 (colonialism or decoloni?ation or empowerment or intersectionality or postcolonialism).tw. 33270
35 ((first generation or special needs) adj3 student*).tw. 3450
36 ((disability* or handicap* or able-ism or ableism or educational or gender or racial) adj3 (discrimination or attitude* or student* or difference*)).tw. 101627
37 (race or racism or antiracism or ethnicity or ((ethnic or minority or racial) adj3 (diversity or group* or attitude*))).tw. 160826
38 (immigration or immigrant* or migrant* or refugee* or paperless).tw. 61995
39 (LGBT* or homosexual* or sexuality or bisexual* or heterosexual* or transsexual* or transgender*).tw. 90206
40 (social class or socioeconomic status or low income student* or social justice or social inclusion or social marginali* or social exclusion or social rejection).tw. 70709
41 (neurodivergen* or neurodivers* or ((mental health or wellbeing or well-being) adj3 (student* or learner* or faculty or teacher* or educator* or instructor* or professor* or staff or train*))).tw. 20112
42 (Religio* or Buddhis* or Christian* or Confucian* or Islam* or Judaism or Hindu* or anthroposoph* or Mormon* or shaman* or spiritualism or taoism or daoism or voodoo or atheis*).tw. 115883
43 or/12-42 785892
44 11 and 43 429
45 limit 44 to ("0200 book" or "0240 authored book" or "0280 edited book" or "0300 encyclopedia") 54
46 44 not 45 375
47 limit 46 to yr="2014 -Current" 212

https://ovidsp.ovid.com/ovidweb.cgi?T=JS&NEWS=N&PAGE=main&SHAREDSEARCHID=5Bmpi3jNZPg3bpxkUPNWHVI7YmkoJjz5EOZ4zt5CyXOOiFOI9jjIdnpBF9oZOLRvN
