# Supplementary material for: Equity, Diversity, Inclusion and Accessibility in Pharmacy Education: A Scoping Review
Source: Pharmacy (Basel). 2026 May 20;14(3):76. doi: 10.3390/pharmacy14030076 (PMC13214706; doi:10.3390/pharmacy14030076)
Supplement: Supplementary file 1 [file pharmacy-14-00076-s001.zip › File S4 Data extraction tool.pdf]

Appendix I

**Data collection form**	
Date of extraction	
**Study identifiers**	
Title	
Author (s) name (last, first)	
Who are the authors in this study (title, job role, associated department)?	
Where are the researchers from (eg, academic institution, organization)?	
Date of publication	
Study country of origin	
Was power, privilege, social locations, or biases acknowledged by the authors when referring to interpretation of these concepts?	□No □Yes Please specify: _______________________
**Eligibility criteria**	
Does this study include EDIA in pharmacy education?<br>□Yes □No	
Which sub question is covered by this study?<br>□Faculty development □Curriculum □Teaching strategies □No	
Is the study abstract text in English?<br>□Yes □No if no, specify language:	
**Study details**	
What was the purpose, aim or objective of this study?	
Was this study peer-reviewed?	□Yes □No
**Methods**	
What was the design?	Quantitative: □Systematic review/meta-analysis □Scoping review □Randomized controlled trial □Cross-sectional study □Cohort study □Other, please specify: Qualitative □Phenomenological □Ethnography □Grounded theory □Narrative inquiry/analysis □Interpretive description □Action research □Case study □Case series □Case-control □Survey □Other, please specify:
**Population**	
What was the population for this study?	Student □Undergraduate □Graduate □Other:<br>□ Staff<br>□Other, please specify: _______________________
**Concept**	
**Faculty development**	
~~Was faculty development addressed?~~	~~□Yes □No~~
What is the EDIA content specificity?	□ Gender □ Language □ Race and ethnicity □ Sexuality □ Disability □ Culture □ Class □ Religion □ First generation □

Appendix I

	Indigenization □ health inequities □ intercultural communication (intercultural competence) □ internationalisation □ decolonisation □Other, please specify: _____________________
Were learning objectives of the course/training content related to EDIA concepts outlined in the paper?	□No □Yes If yes, what were they?
**Curriculum**	
What is the EDIA content specificity?	□ Gender □ Language □ Race and ethnicity □ Sexuality □ Disability □ Culture □ Class □ Religion □ First generation □ Indigenization □ Health inequities □ Intercultural communication □ Internationalization □ Illiteracy □ Decolonization □ Other, please specify: _____________________
Impetus or process for curriculum development	□International guidelines □National guidelines □Professional entity guidelines □ Institutional guidelines □ Community academia collaboration □Other, please specify: _____________________
**Teaching strategies**	
What pharmacy specific subject is addressed?	□Social pharmacy □Clinical Pharmacy □Pharmacology □Pharmacy practice □Pathophysiology □Biopharmaceutics □Pharmaceutical chemistry □ Undefined □Other, please specify: _____________________
What is the EDIA content specificity?	□ Gender □ Language □ Race and ethnicity □ Sexuality □ Disability □ Culture □ Class □ Religion □ First generation □ Indigenization □ Health inequities □ Intercultural communication □ Internationalization □ Illiteracy □ Decolonization □ Other, please specify: _____________________
**Context**	
**Faculty development**	
What was the mode of delivery?	□Single session □ Multi-session □Self-directed □Experiential learning □Other, please specify: _____________________
Was it mandatory?	□ Yes □ No □ Not specified
How was it delivered?	Fully online □ fully in person □Combination
What was the style of delivery?	□Lecture-based □Discussion-based □Activity-based □ Simulation based □ Immersive □Other, please specify: _____________________
What were the learning activities?	□Video/podcast □Readings/articles □Case-based □Small group discussion □Large group discussion □Role play □Reflection based □Other, please specify: _____________________
What was the total time investment (In hours)?	□ 1-3 □ 4-10 □ 11- 20 □ 20+ □ Not specified
Who developed the curricular content?	□ Pharmacy □ Institution □ Government □ University pedagogy □Professional organization □EDIA specialists □ students □ undefined □Other, please specify: _____________________
Were pharmacists involved?	□ No □ Yes if yes, in: □ Development and design □ Implementation □ Evaluation □ Not specified
Were the concepts/learning objectives assessed (eg, outcome measures)?	□ Yes □ No □ Not specified

Did the authors provide specific recommendations?	□No □Yes Please specify: ______________________
Did the authors recommend a specific framework, guideline, model, and/or curriculum?	□No □Yes: □Framework □Guideline □Model □Curriculum □Other: ______________________ If yes, please explain which framework/guideline/model used: ____
**Curriculum**	
What was developed?	□Standalone course, elective □Standalone course, mandatory □Embedded in broader courses, elective □Embedded in broader courses, mandatory □ Seminars/Webinars □Other, please specify: ______________________
How is it delivered?	□Fully online □ fully in person □Combination
In addition to pharmacists, who developed it?	□ Institution □ University pedagogy □Professional organization □EDIA specialists □ students □ undefined □Other, please specify: ______________________
Were the concepts/learning objectives assessed (eg, outcome measures)?	□ Yes □ No □ Not specified
Did the authors provide specific recommendations?	□No □Yes Please specify: ______________________
Did the authors recommend a specific framework, guideline, model, and/or curriculum?	□No □Yes: □Framework □Guideline □Model □Curriculum □Other: ______________________ If yes, please explain which framework/guideline/model used: ____
**Teaching strategies**	
What is the format of teaching strategies?	□Traditional lecturing □ Experiential Learning □Team-based learning (TBL) □ Problem-based learning (PBL) □ Role play (counselling, communication) □ Simulation-based learning □ Community engaged learning □ Peer Feedback □Assessment □Other, please specify: ______________________
How was it delivered?	Fully online □ fully in person □Combination
In addition to the pharmacy educator, who developed it?	□ Institution □ University pedagogy □ Professional organization □ EDIA specialists □ Students □ Community representative □ undefined □Other, please specify: ______________________
How were the concepts/learning objectives assessed (eg, outcome measures)?	□ Yes □ No □ Not specified
Did the authors provide specific recommendations?	□No □Yes Please specify: ______________________
Did the authors recommend a specific framework, guideline, model, and/or curriculum?	□No □Yes: □Framework □Guideline □Model □Curriculum □Other: ______________________ If yes, please explain which framework/guideline/model used: ____
**Limitations**	

What were the limitations of this study?	
